# Nursing care pathways for sustainable and healthy aging

**DOI:** 10.1590/0034-7167-2024-0051

**Published:** 2025-06-13

**Authors:** Daiane de Souza Fernandes, Elisa Rosas Cervantes, Mauriely Paiva de Alcântara e Silva, Rosalina Aparecida Partezani Rodrigues

**Affiliations:** I Universidade de São Paulo. Ribeirão Preto, São Paulo, Brazil; II Universidad de Guanajuato. Celaya, Guanajuato, Mexico

**Keywords:** Nursing, Geriatric Nursing, Aged, Healthy Aging, Sustainable Development., Enfermería, Enfermería Geriátrica, Anciano, Envejecimiento Saludable, Desarrollo Sostenible.

## Abstract

**Objectives:**

to reflect on the alignment between the four action areas of the Decade of Healthy Ageing and the Sustainable Development Goals, analyzed in light of the nursing care pathway for older adults.

**Methods:**

a reflective essay based on the Sustainable Development Goals and the Decade of Healthy Ageing documents, guided by the concepts of nursing care pathways for older adults.

**Results:**

the reflection included 11 of the 17 Sustainable Development Goals related to the four areas investigated and nursing care pathway: Older adults, Family, Nursing care, and Nursing research.

**Final Considerations:**

the nursing care pathway for older adults, as aligned with the principles of policies for sustainable and healthy aging, enables nurses to lead the process of ensuring a more dignified life for the older population.

## RESUMEN

**Objetivos:**

reflexionar sobre la alineación entre las cuatro áreas de acción de la Década del Envejecimiento Saludable y los Objetivos de Desarrollo Sostenible, analizados desde la perspectiva de las líneas de cuidado de enfermería dirigidas a las personas mayores.

**Métodos:**

ensayo de carácter reflexivo basado en los documentos de los Objetivos de Desarrollo Sostenible y de la Década del Envejecimiento Saludable, siguiendo las concepciones de las líneas de cuidado de enfermería para personas mayores.

**Resultados:**

la reflexión incluyó 11 de los 17 Objetivos de Desarrollo Sostenible relacionados con las cuatro áreas investigadas y linea de cuidado: Persona mayor, Familia, Cuidado de enfermería e Investigación en enfermería.

**Consideraciones Finales:**

Las líneas de cuidado dirigidas a las personas mayores, conforme a los principios de las políticas para un envejecimiento sostenible y saludable, permiten a los profesionales de enfermería liderar el proceso de garantizar una vida más digna para la población mayor.

## INTRODUCTION

Aging is a determinant phenomenon of the well-being of any population, with implications spanning labor, economic, governmental, family, societal, and healthcare system spheres. Over the years, leaders worldwide have sought to ensure the health and well-being of older people, given that they represent the fastest-growing age group today^(1)^.

In 2015, to eradicate poverty, protect the planet, and ensure peace and prosperity for all, the United Nations adopted the 17 Sustainable Development Goals (SDGs) to be achieved by 2030^(2)^. Subsequently, the World Health Organization (WHO), during the 2016 World Health Assembly, proposed strategies to incorporate the SDGs into health-related initiatives, granting significant attention to aging. From this arose the concept of *Healthy Ageing*, defined as a process of actions and strategies to develop and preserve functional ability and intrinsic capacity, as well as improve the environments in which older people thrive to ensure overall well-being during this life stage^(1,3)^.

Based on these strategies, planning efforts were directed toward a ten-year period termed the *Decade of Healthy Ageing* (DHA), encompassing 2020 to 2030. Its goal is to impact social and health sectors to promote and maintain health, prevent diseases, and preserve the functional ability and intrinsic capacity of older people.

For this purpose, four action areas were established: 1) Changing how we think, feel, and act regarding age and aging; 2) Ensuring communities foster the abilities of older people; 3) Delivering person-centered, integrated care and Primary Health Care (PHC) services tailored to older people; and 4) Providing access to long-term care for older people who need it^(3)^.

Implementing these areas in daily practice represents a challenge for health professionals, as care for older people must be structured differently from that for other age groups, with its primary focus on health rather than disease. Nurses, committed to advancing the SDGs and the DHA for the benefit of older people, integrate specific care pathways (CPs) tailored to this population. They aim to act in different contexts and areas of the Decade to ensure sustainable aging.

The *nursing care pathways* for older people are standardized care strategies supported by the multidisciplinary team. The goal is to promote health, prevent harm, treat, and rehabilitate diseases for older people seeking care within a Health Care Network (HCN)^(4)^. However, these pathways have not been effectively implemented in the routine care of older people. Moreover, the programs executed in practice have often been disease-focused rather than health-oriented. In contrast, care for older people in society should be specifically structured, considering the physiological changes requiring greater attention for this group.

Given the above and the grounding in the SDGs, the DHA, the CP model, and the complexity of the human being, a four-pillar approach for nursing care for older people is proposed, as follows: 1) *Older individual* refers to someone aged 60 or older requiring health attention; 2) *Family* of the older individual is defined as the group, whether related by blood or not, deemed essential by the individual for their care and coexistence; 3) *Nursing care* encompasses practices developed by nursing teams for older people in various contexts and needs, aiming to maintain healthy aging while directing actions toward policy formulation and decision-making; and 4) *Nursing research* is conducted by nurses within the four action areas to address the needs of older people and impact health services, thereby fostering greater participation in local and global decision-making processes^(5)^.

The authors of this study are nurses and researchers in the field of aging. Considering the SDGs and the DHA, nursing will be expected to move toward reflecting on how to advance proposals aligned with the principles of these policies and nursing care pathways for older people.

## OBJECTIVES

To reflect on the alignment between the four action areas in the Decade of Healthy Ageing and the Sustainable Development Goals, considered in light of the nursing care pathway for older people.

## METHODS

This is a reflective essay. To achieve the objective, we used the documents of the Sustainable Development Goals^(2)^ and the Decade of Healthy Aging^(3)^, analyzed from the perspective of the nursing care pathway conceptions^(4,5)^. These documents demonstrate how nursing can adjust care actions for older people based on these frameworks to promote sustainable aging.

Data collection began with an initial reading of the aforementioned documents. Subsequently, cross-mapping was employed to link and group the proposals based on each document, thus composing the final reflection on planning the four action areas that nursing must incorporate into its praxis.

## RESULTS

With demographic and epidemiological changes worldwide, especially in developing countries, the SDGs and DHA have spurred debates on the global aging process. These discussions are grounded in published documents^(6)^ and other global and national public policies aimed at ensuring dignity and greater attention to older people across various social segments.

In this context, nursing holds a prominent role in society, given these changes and the increasing demand for care for older people across different levels of care. This new social challenge highlights the need for nursing to direct its clinical practice and competencies toward models unique to the profession, particularly the nursing care pathway, which addresses the specific needs of this population. Reflections on the experiences developed so far allow for evaluating how practice should align with these frameworks and for reporting possibilities for advancing care through innovative proposals.

It is worth noting that global discourses promoted by entities such as the United Nations foster the creation of moral compasses for aging. A first example is the DHA, which serves as a catalyst and collaborative action to improve the lives of older people, families, and communities in the face of current challenges. A second example is the SDGs, which establish a set of values that provide a moral guide for the necessary advancements^(7)^.

As a result of the cross-mapping conducted by the study authors between the 17 goals of the Sustainable Development Goals^(2)^ and the four action areas of the Decade of Healthy Aging^(3)^, 11 were found to be aligned: 1) No poverty; 2) Zero hunger; 3) Good health and well-being; 4) Quality education; 5) Gender equality; 8) Decent work and economic growth; 9) Industry, innovation, and infrastructure; 10) Reduced inequalities; 11) Sustainable cities and communities; 16) Peace, justice, and strong institutions; and 17) Partnerships for the goals. These goals were linked to the four nursing care pathways: 1) Older people; 2) Family; 3) Nursing care; and 4) Nursing research.

**Figure 1:**
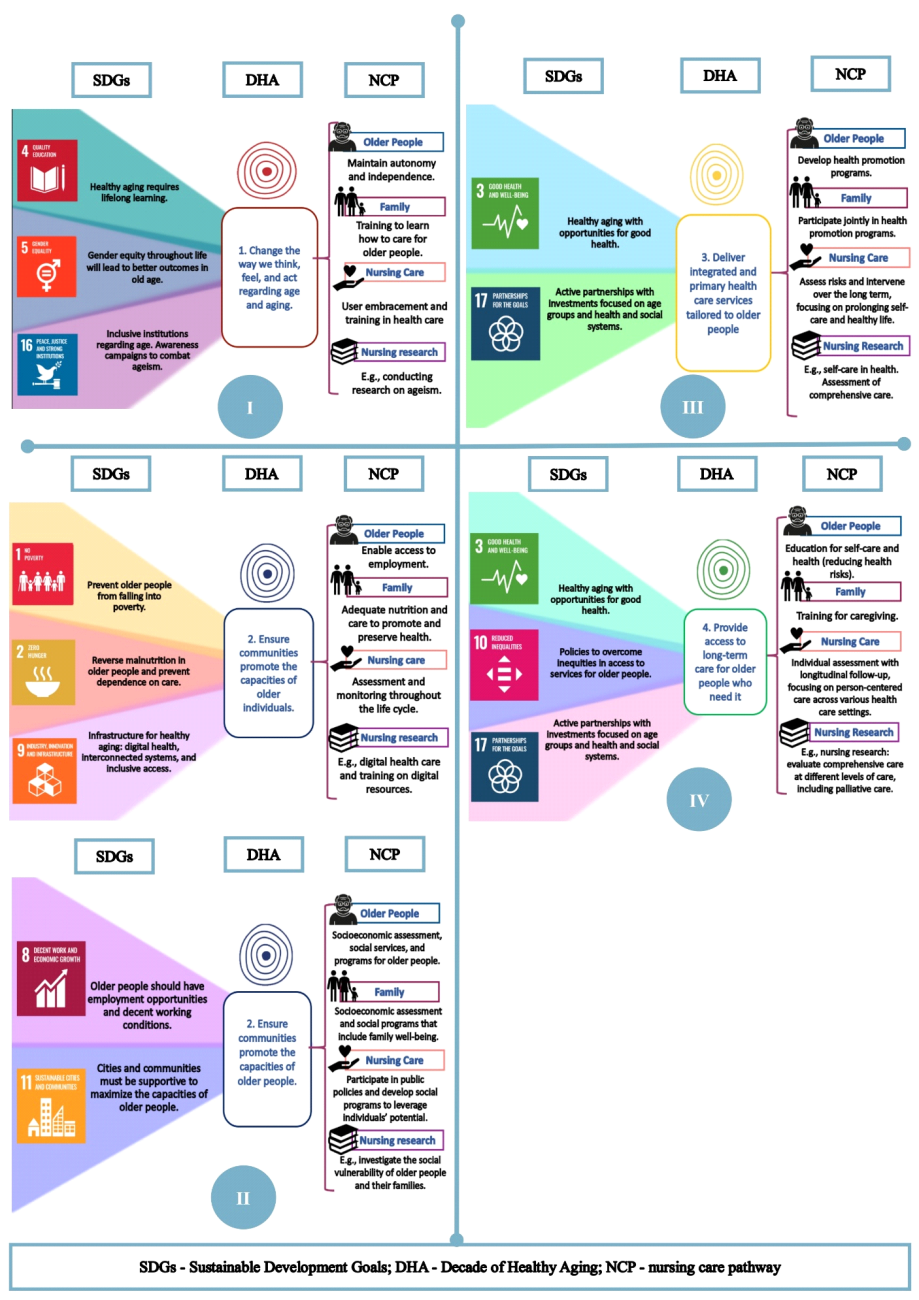
Alignment of the Sustainable Development Goals with the Decade of Healthy Aging, analyzed from the perspective of the nursing care pathway, Ribeirão Preto, São Paulo, 2024

*Source: Sustainable Development Goals and Decade of Healthy Aging, adapted by the authors to include nursing care pathways for older people, Ribeirão Preto, São Paulo, 2024.*

To facilitate understanding of this document analysis from the perspective of the nursing care pathway for older people, Figure 1 presents intervention proposals that enable reflections focused on sustainable and person-centered nursing care. The same figure includes ideas to be applied to the clinical practice of the nursing care pathway for each goal. For example, in action area III for older people, the development of health promotion programs is suggested. Next, the proposed alignment between the SDGs, DHA, and nursing care pathways is detailed.

### Changing the way we think and feel about aging

In analyzing the action area aimed at changing the way we think and feel about aging, we identified that this action aligns with items 4, 5, and 16 of the SDGs, while the nursing care pathways focus on educating older people to maintain their autonomy and independence. In this regard, encouraging active participation of older people in their health and care decisions is essential for maintaining health.

Nursing care pathways must focus on developing specific actions aimed at: maintaining the autonomy and independence of older people; educating families about the aging process; and delivering care during this new phase of life. Education for older people and their families should occur in various healthcare settings, always keeping in mind that this is the main focus, despite the vast majority still lacking access to healthcare services.

SDGs 5 and 16 are aligned in this context. This is because gender equity throughout the life course as a strategy for achieving better outcomes in old age, age-inclusive institutions, and awareness campaigns against prejudice are fundamental for society to reframe its perspective on aging.

It is also essential to raise awareness among nurses about promoting active and healthy aging through scientific research, with an emphasis on studies addressing ageism. Furthermore, nursing’s efforts to combat this type of prejudice contribute to developing policies and practices that fight discrimination and ensure equitable access to high-quality healthcare for this population. In this context, the nurse’s role is essential in fostering a culture that values and respects older people, thereby contributing to the creation of a more inclusive society^(8)^.

It is important to highlight that prejudice against aging marginalizes older people and limits their protagonism within families, society, and communities. The sociocultural construction of aging as a process of ostracizing older people must be actively combated. In everyday practice, it is necessary to foster social bonds that encourage a focus on the ability of this older population to “be” and “do”, in favor of their well-being.

From this perspective, nursing plays a significant role in the continuing education of healthcare professionals, aiming to connect the labeling of older people with the consequences of age-based prejudice and emphasizing the importance of this population group in social interactions.

### Ensuring the promotion of older people capacities

The second action area focuses on fostering the maintenance of capacities emphasizing physical, social, and economic environments. Additionally, it aligns with the following SDGs: 1) Highlights the need to prevent older people from living in poverty; 2) Focuses on nutrition, promoting actions to prevent and control malnutrition, thereby avoiding dependence on care; 8) Underscores the importance of providing older people with employment opportunities and dignified working conditions; 9) Emphasizes the need for infrastructure to support healthy aging, particularly through digital health and access to inclusive technologies; and 11) Stresses the value of cities and communities that are conducive to maximizing older people capacities.

As this action area is directly related to the environment, it reaffirms that physical, social, and economic environments often either hinder or facilitate healthy and sustainable aging. It should be clear that aging with quality of life requires aligning intrinsic capacity (physical and mental abilities) with the environment. Thus, global environmental changes can foster the inclusion of older populations and reduce inequalities^(7)^.

The various types of environments an individual passes throughout their life significantly influence their life trajectory. In old age, the ability to “age in place”-preserving autonomy, aging with nutritional and social dignity, equitable access to services, and respect for individual and community-specific needs-can contribute to sustainable and healthy aging.

Maintaining functional ability is facilitated by developing nursing care pathways that encourage employment opportunities for older people, provide environments adapted to their needs, ensure access to proper nutrition, and promote health and disease prevention through integrated, needs-centered evaluations and follow-up care.

From this perspective, the physical or social environment where an older individual is situated significantly impacts their functional ability, either by creating barriers or providing stimuli that shape capacities, opportunities, choices, and behaviors. Furthermore, these environments are directly associated with intrinsic capacity^(4)^.

The importance of nurses’ involvement in formulating and implementing public policies is also emphasized. This includes developing programs to empower this population and encouraging research on topics such as digital health, monitoring age-friendly environments, and addressing social vulnerability. It is urgent to conduct broad investigations into this subject, as social vulnerability compromises physical and mental health and increases frailty. Therefore, addressing healthy aging requires evaluating vulnerability to predict the risks of adverse health and living conditions for older populations.

### Providing integrated and primary care centered on older people

This third action aligns with the following SDGs: 3) Highlights healthy aging as essential for good health, and 17) Emphasizes the need for active partnerships with favorable investments for age groups within health and social systems.

Person-centered care in PHC refers to an approach that places the older individual at the core of the care process, recognizing their individual needs, preferences, values, and goals. Additionally, it facilitates the promotion of an integrated and coordinated approach among the various health professionals involved, reducing care fragmentation^(9)^. In this sense, the provision of integrated and person-centered PHC represents a fundamental pillar for the health and well-being of older people. The intersection of environment, life course, and well-being reflects on healthy and sustainable aging, reinforcing the importance of ensuring access to essential services for the older population to maintain this pillar. It is also essential to note that health systems require changes to incorporate metrics for healthy aging.

In this regard, nursing care pathways suggest actions such as implementing health promotion and disease prevention programs at the primary level, emphasizing the importance of family involvement in these initiatives. Person-centered nursing care in various care areas and reinforcing the significance of comprehensive care assessments across all domains-such as conducting multidimensional assessments-can contribute to successful and sustainable aging.

Research conducted by nurses on topics like self-care and the evaluation of comprehensive care can guide the development of more effective strategies to promote the well-being of the older population.

### Promoting access to long-term care for older people

The fourth action area highlights the importance of promoting access to long-term care and is aligned with the following SDGs: 3) Highlights the importance of healthy aging for maintaining health and well-being; 10) Emphasizes the need for public policies to address inequities in service access for older populations; and 17) Stresses the importance of partnerships with favorable investments for age groups.

Older people of advanced age may experience declines in functional ability, often manifested as cognitive, physical, and social changes that may occur either in isolation or concomitantly, necessitating long-term care. In this context, nursing leads the provision of typified, comprehensive, and person-centered care across various settings, such as long-term care facilities, rehabilitation centers, and homes.

Thus, implementing health promotion programs focused on self-care for older people and their families and aligning a longitudinal plan for comprehensive care are essential for applying nursing care pathways. Nurses play a vital role in educating patients and their families, as well as in health promotion and prevention measures, operating under a broader understanding of health^(10)^.

Additionally, research and subsequent publications are crucial to ensure findings reach society, including older people. In this proposition, nurses should act as leaders in public policy formulation, advocating for comprehensive care across all levels of care. This includes PHC for healthy older people and palliative care. Studies on comprehensive and longitudinal care across different levels of care can guide healthcare teams, ensuring older populations gain access to longterm care.

Nursing professionals are closest to the public and, as such, are capable of effectively managing care processes. It is important to emphasize that to achieve healthy and sustainable aging, nurses must collaborate with interdisciplinary teams, adopting a multidimensional view of older people when developing integrative actions.

However, some countries lack effective management to implement changes and value older people, taking into account the particularities of each social setting. This is an ambitious proposal. In this sense, the United Nations, the World Health Organization, the Pan American Health Organization, universities, and professional councils must debate this issue and propose changes to the roles of nurses.

## FINAL CONSIDERATIONS

The Decade of Healthy Ageing and the Sustainable Development Goals, considered from the perspective of nursing care pathway concepts, provide a framework to reflect on how nurses can and should play a role in implementing strategies that foster successful and sustainable aging. Therefore, the realization of the propositions presented in this reflective analysis emerges as one of the major challenges for advancing gerontological nursing.
