# Supplementary material for: Nursing care pathways for sustainable and healthy aging
Source: Rev Bras Enferm. 2025 Jun 13;78(Suppl 1):e20240051. doi: 10.1590/0034-7167-2024-0051 (PMC12165716; doi:10.1590/0034-7167-2024-0051)
Supplement: Translation

# Translation: Linhas de cuidado de enfermagem para o envelhecimento sustentável e saudável

**DOI:** 10.1590/0034-7167-2024-0051pt

**Authors:** Daiane de Souza Fernandes, Elisa Rosas Cervantes, Mauriely Paiva de Alcântara e Silva, Rosalina Aparecida Partezani Rodrigues

**Affiliations:** I Universidade de São Paulo. Ribeirão Preto, São Paulo, Brasil; II Universidad de Guanajuato. Celaya, Guanajuato, México

**Keywords:** Enfermagem, Enfermagem Geriátrica, Idoso, Envelhecimento Saudável, Desenvolvimento Sustentável.

## Abstract

**Objetivos:**

refletir sobre o alinhamento entre as quatro áreas de ação da Década do Envelhecimento Saudável e os Objetivos do Desenvolvimento Sustentável, analisados à luz da linha de cuidado de enfermagem às pessoas idosas.

**Métodos:**

ensaio do tipo reflexivo com base nos documentos dos Objetivos do Desenvolvimento Sustentável e da Década de Envelhecimento Saudável, seguindo as concepções da linha de cuidado de enfermagem às pessoas idosas.

**Resultados:**

a reflexão incluiu 11 dos 17 Objetivos do Desenvolvimento Sustentável relacionados com as quatro áreas investigadas e linhas de cuidado: Pessoa idosa, Família, Cuidado de enfermagem e Pesquisa em enfermagem.

**Considerações Finais:**

a linha de cuidado da pessoa idosa, conforme os pressupostos das políticas para envelhecimento sustentável e saudável, permite aos enfermeiros liderar o processo de garantir uma vida mais digna à população idosa.

## INTRODUÇÃO

O envelhecimento é um fenômeno determinante do bem-estar de qualquer população, com implicações desde a esfera laboral, econômica, governamental até o funcionamento da família, da sociedade e de todo o sistema de saúde. Por isso, ao longo dos anos, líderes de todos os países têm se preocupado em garantir a saúde e o bem-estar das pessoas idosas, uma vez que representam a faixa etária em maior expansão atualmente^(1)^.

Em 2015, com a esperança de erradicar a pobreza, proteger o planeta e garantir a paz e a prosperidade para todos, as Nações Unidas adotaram os 17 Objetivos de Desenvolvimento Sustentável (ODS) a serem alcançados até 2030^(2)^. A Organização Mundial da Saúde (OMS), por sua vez, na Assembleia Mundial da Saúde de 2016, propôs uma série de estratégias para trabalhar os ODS no campo da saúde, passando a conferir um importante papel ao envelhecimento. Desde então, emergiu o conceito de “Envelhecimento Saudável”, definido como um processo de trabalho e ações que permite desenvolver e preservar a capacidade funcional, a capacidade intrínseca e melhorar o ambiente onde as pessoas idosas se desenvolvem, para assegurar o bem-estar geral durante essa fase de vida^(1,3)^.

Com base nessas estratégias, o planejamento passou a concentrar esforços voltados a um período de dez anos, denominado “Década do Envelhecimento Saudável” (DES), compreendida de 2020 a 2030. O objetivo é impactar setores sociais e de saúde que desencadeiam a promoção e manutenção da saúde, prevenção de doenças, preservação da capacidade funcional e da capacidade intrínseca das pessoas idosas.

Para tanto, foram estabelecidas quatro áreas de atuação: 1) Mudar a maneira como pensamos, sentimos e agimos com relação à idade e ao envelhecimento; 2) Garantir que comunidades promovam as capacidades das pessoas idosas; 3) Entregar serviços de cuidados integrados e de Atenção Primária à Saúde (APS) centrados no indivíduo e adequados à pessoa idosa; e 4) Propiciar acesso a cuidados de longo prazo às pessoas idosas que necessitarem^(3)^.

A inserção dessas áreas de atuação na prática diária representa um desafio para os profissionais de saúde em geral, pois o cuidado a pessoas idosas deve ser estruturado de forma diferente daquele prestado a outras faixas etárias e seu foco principal deve ser a saúde e não a doença. Os profissionais de enfermagem, comprometidos com a promoção dos ODS e a DES em benefício das pessoas idosas, integram-se às linhas de cuidado (LC) específicas a essa população. Eles buscam atuar em diferentes contextos e em áreas de ação da Década, a fim de garantir um envelhecimento sustentável.

As “linhas de cuidado de enfermagem” para pessoas idosas são estratégias de cuidado padronizadas pela área e apoiadas por toda a equipe multidisciplinar. O objetivo é realizar ações de promoção da saúde e prevenção de agravos, tratamento e reabilitação de doenças para a população idosa que procura atendimento em uma Rede de Atenção à Saúde (RAS)^(4)^. Entretanto, essas linhas não têm sido efetivamente realizadas no cotidiano do cuidado da pessoa idosa. Além disso, as propostas de programas efetivadas na prática têm tido relação com a doença e não com a saúde. Em contraste, os cuidados de pessoas idosas na sociedade deveriam ser estruturados de forma específica, dadas as mudanças fisiológicas que requerem maior atenção a esse grupo.

Diante do exposto e da fundamentação nos ODS, na DES, no modelo de LC e na consideração da complexidade do ser, propõe-se uma abordagem de quatro eixos para o cuidado de enfermagem de pessoas idosas, a saber: 1) “Pessoa idosa” é considerada aquela com 60 anos ou mais, que necessita de atenção à saúde; 2) “Família” da pessoa idosa é aquele grupo, consanguíneo ou não, tido por ela essencial para sua convivência e cuidado; 3) “Cuidado de enfermagem” é a prática desenvolvida pela equipe de enfermagem às pessoas idosas em diferentes contextos e necessidades, visando manter um envelhecimento saudável e direcionando suas ações para níveis de formulação de políticas e tomada de decisão; e 4) “Pesquisa em enfermagem” é desenvolvida pelos enfermeiros em cada uma das quatro áreas de ação, para responder às necessidades das pessoas idosas e impactar os serviços de saúde, proporcionando, assim, maior participação na tomada de decisão local e global^(5)^.

Os autores deste estudo são enfermeiros e pesquisadores da área do envelhecimento. Considerando os ODS e a DES, espera-se que a enfermagem caminhe no sentido de refletir sobre como podemos avançar em propostas tangenciadas com os princípios dessas políticas e linhas de cuidado de enfermagem para a pessoa idosa.

## OBJETIVOS

Refletir sobre o alinhamento entre as quatro áreas de ação da Década do Envelhecimento Saudável e os Objetivos do Desenvolvimento Sustentável, considerados à luz da linha de cuidado de enfermagem para pessoas idosas.

## MÉTODOS

Trata-se de um ensaio do tipo reflexivo. Para alcançar o objetivo, foram utilizados os documentos dos Objetivos do Desenvolvimento Sustentável^(2)^ e da Década de Envelhecimento Saudável^(3)^, analisados sob a perspectiva das concepções da linha de cuidado de enfermagem^(4,5)^. Tais documentos permitem mostrar como a enfermagem pode ajustar as ações do cuidado à pessoa idosa com base nesses referenciais, a fim de promover o envelhecimento sustentável.

A coleta de dados iniciou-se com uma leitura geral dos documentos já elencados. Em seguida, utilizou-se o mapeamento cruzado para ligar e agrupar a proposta baseada em cada documento, compondo, assim, a reflexão final do planejamento das quatro áreas de ação que a enfermagem deverá incorporar na sua práxis.

## RESULTADOS

Com as mudanças demográficas e epidemiológicas no mundo, sobretudo nos países em desenvolvimento, os ODS e a DES vêm suscitando debates acerca do processo de envelhecimento mundial. Essas discussões baseiam-se em documentos já publicados^(6)^ e em outras políticas públicas mundiais e nacionais para garantir a vida da pessoa idosa com dignidade e maior atenção, nos diferentes segmentos sociais.

Nesse contexto, a enfermagem ocupa espaço de destaque na sociedade em face dessas mudanças e do maior número de atendimento de pessoas idosas nos diferentes níveis de atenção. Esse novo desafio social revelou a necessidade de a enfermagem direcionar sua prática clínica/competências para modelos próprios da profissão, sobretudo na LC de enfermagem, a qual atende às peculiaridades da população. As reflexões sobre as experiências desenvolvidas até o momento permitem tanto avaliar como deve ser a prática com base nesses referenciais em conjunto quanto relatar possibilidades de avanço nesse cuidado mediante propostas desafiadoras.

Convém destacar que discursos globais promovidos por entidades como as Nações Unidas propiciam criar bússolas morais para o envelhecimento. Um primeiro exemplo é a DES, que atua como uma ação catalisadora e colaborativa para melhorar a vida da população idosa, famílias e comunidades perante os desafios vivenciados. Um segundo exemplo são os ODS, por estabelecerem um conjunto de valores que fornece um guia moral para os necessários avanços^(7)^.

Como resultado do cruzamento que os autores do estudo realizaram dos 17 eixos dos Objetivos do Desenvolvimento Sustentável^(2)^ com as quatro áreas de ação da Década do Envelhecimento Saudável^(3)^, 11 mostraram-se alinhados: 1. Fim da pobreza: 2. Fome zero; 3. Saúde e bemestar; 4. Educação de qualidade; 5. Igualdade de gênero; 8. Trabalho decente e crescimento econômico; 9. Indústria, inovação e infraestrutura; 10. Redução das desigualdades; 11. Cidades e comunidades saudáveis; 16. Paz, justiça e instituições sólidas; e 17. Alianças para atingir os objetivos. Esses eixos foram relacionados com as quatro linhas de cuidado: 1. Pessoa Idosa; 2. Família; 3. Cuidado de enfermagem; e 4. Pesquisa em enfermagem.

Para facilitar a compreensão dessa análise dos documentos sob a ótica da linha de cuidado de enfermagem para a pessoa idosa, a Figura 1 apresenta as propostas de intervenção que permitem reflexões voltadas a um cuidado de enfermagem centrado e sustentável. Na mesma figura, expõem-se ideias a serem utilizadas na prática clínica da LC em cada eixo. Por exemplo, na área de ação III para a pessoa idosa, sugere-se o desenvolvimento de programas de promoção à saúde. A seguir, detalhase o alinhamento proposto entre os ODS, DES e LC.

**Figura 1:** Alinhamento os Objetivos do Desenvolvimento Sustentável com as áreas da Década do Envelhecimento Saudável, analisados à luz da linha de cuidado de enfermagem, Ribeirão Preto, São Paulo, 2024

*Fonte: Objetivos do Desenvolvimento Sustentável e Década do Envelhecimento Saudável, adaptados pelas autoras com inclusão das linhas de cuidado para a pessoa idosa, Ribeirão Preto, São Paulo, 2024.*

### Mudar a maneira como pensamos e sentimos o envelhecimento

Na análise da ação para mudar a forma como pensamos e sentimos o envelhecimento, identificou-se que essa ação relaciona-se com os itens 4, 5 e 16 do ODS, enquanto as LC se direcionam à educação da pessoa idosa para que ela mantenha sua autonomia e independência. Nesse sentido, o incentivo à participação ativa das pessoas idosas nas decisões relacionadas à sua saúde e cuidado é essencial para a manutenção da saúde.

As LC de enfermagem devem estar focadas no desenvolvimento de ações específicas voltadas para: a manutenção da autonomia e independência das pessoas idosas; a capacitação das famílias sobre o processo de envelhecimento; e o cuidado nessa nova fase de vida. A educação das pessoas idosas e seus familiares deve ocorrer nos diferentes espaços de cuidado à saúde, lembrando sempre que este é o foco, embora a grande maioria ainda não tenha acesso aos serviços de saúde.

Os ODS 5 e 16 estão alinhados. Isso porque a equidade de gênero no curso de vida como estratégia para obter melhores resultados na velhice, as instituições inclusivas quanto à idade e as campanhas de conscientização contra o preconceito são fundamentais para que a sociedade ressignifique o envelhecimento.

É também primordial sensibilizar o enfermeiro para a promoção de um envelhecimento ativo e saudável por meio da investigação científica, com destaque para pesquisas, como aquelas sobre o idadismo. Ademais, a atuação da enfermagem contra esse tipo de preconceito auxilia na promoção de políticas e práticas que combatem a discriminação e garantem o acesso igualitário aos cuidados de saúde de qualidade para essa população. Nesse contexto, o papel do enfermeiro é essencial na promoção de uma cultura que valoriza e respeita as pessoas idosas, contribuindo para a construção de uma sociedade mais inclusiva^(8)^.

Importante salientar que o preconceito em relação à idade marginaliza e limita o protagonismo da pessoa idosa na família, sociedade e comunidade. A construção sociocultural em torno do envelhecimento como um processo de ostracismo das pessoas idosas necessita ser combatida. Na prática cotidiana, precisa ser vivenciado o incentivo à constituição de vínculos sociais que estimulem o foco na capacidade dessa população idosa de “ser” e fazer”, em prol do bem-estar.

Dessa perspectiva, a enfermagem desempenha um papel relevante na educação permanente de profissionais de saúde, no intuito de relacionar rótulos envolvendo a população idosa e as consequências do preconceito contra a idade, enfatizando a importância desse grupo populacional no convívio social.

### Garantir a promoção das capacidades das pessoas idosas

A segunda área de ação está focada no estímulo à manutenção das capacidades com enfoque nos ambientes físicos, sociais e econômicos. Além disso, apresenta conexão com os seguintes ODS: 1. Enfatiza a necessidade de se evitar que as pessoas idosas vivam na pobreza; 2. Enfoque na nutrição, com desenvolvimento de ações para prevenir e controlar a desnutrição, portanto evitando a dependência de cuidados; 8. Ressalta a importância de a pessoa idosa ter oportunidades de emprego e condições dignas de trabalho; 9. Destaca a necessidade de infraestrutura para um envelhecimento saudável, com ênfase na saúde digital e acesso a tecnologias inclusivas; e 11. Evidencia o mérito das cidades e comunidades favoráveis a maximizar as capacidades da pessoa idosa.

Por se tratar de uma área diretamente relacionada com o meio ambiente, ratifica-se que os ambientes físicos, sociais e econômicos podem, muitas vezes, impedir ou estimular o envelhecimento saudável e sustentável. Deve ficar claro que, para se envelhecer com qualidade de vida, é necessário um ajuste da capacidade intrínseca (habilidades físicas e mentais) com o ambiente. Portanto, mudanças globais no ambiente podem permitir a inclusão da população idosa e a redução de desigualdades^(7)^.

Os diferentes tipos de ambiente pelos quais uma pessoa perpassou durante sua trajetória de vida influenciam seu curso de vida. Na velhice, “ficar onde se quer*”* - com a preservação da autonomia, envelhecendo com dignidade nutricional, social, equidade de acesso aos serviços e respeito às peculiaridades individuais e comunitárias - pode contribuir para um envelhecimento sustentável e saudável.

A manutenção da capacidade funcional é favorecida pelo desenvolvimento de LC que estimulem a promoção do trabalho para a pessoa idosa, o ambiente adequado às suas necessidades, a garantia de acesso à nutrição adequada e aos cuidados de promoção da saúde e a prevenção de doenças mediante avaliações e acompanhamento integrados e centrados nas necessidades da pessoa idosa.

Sob essa ótica, o ambiente onde a pessoa idosa está inserida, seja físico, seja social, impacta a sua capacidade funcional, pela possibilidade de estabelecer barreiras ou estímulos que moldam as capacidades, oportunidades, escolhas e comportamentos. Além disso, tal ambiente tem relação com a capacidade intrínseca da pessoa idosa^(4)^.

Também ressalta-se a importância de envolvimento do enfermeiro na formulação e implementação de políticas públicas, com destaque tanto para o desenvolvimento de programas que estimulem o empoderamento desta população quanto para o incentivo de pesquisas relacionadas a temas como saúde digital, monitoramento de ambientes do tipo Amigo do Idoso e atenção à vulnerabilidade social. Urge investigar este tema amplamente, pois a vulnerabilidade social leva ao comprometimento da saúde física e mental e à fragilidade. Para abordar o envelhecimento saudável, deve-se, portanto, avaliar a vulnerabilidade para predizer os riscos de efeitos adversos da saúde e condição de vida da população idosa.

### Prestar cuidados integrados e cuidados primários centrados na pessoa idosa

Esta terceira ação apresenta encadeamento com os seguintes ODS: 3. Enfatiza o envelhecimento saudável para uma boa saúde; e 17. Reforça a demanda por parcerias ativas com investimentos favoráveis para grupos etários nos sistemas de saúde e social.

O cuidado centrado na pessoa idosa na APS refere-se a uma abordagem que a posiciona no centro do processo de cuidado, reconhecendo suas necessidades, preferências, valores e metas individuais. Adicionalmente, possibilita a promoção de uma abordagem integrada e coordenada entre os diversos profissionais de saúde envolvidos nesse cuidado, o qual se torna menos fragmentado^(9)^. Nesse sentido, a prestação de cuidados integrados e centrados na APS constitui um pilar fundamental para a saúde e bem-estar da pessoa idosa. A junção do ambiente, curso de vida e bem-estar tem reflexos no envelhecimento saudável e sustentável, o que reforça a importância de assegurar a disponibilidade de acesso a serviços essenciais para a população idosa, com vistas à manutenção desse pilar. Ressalta-se também serem necessárias mudanças nos sistemas de saúde para incorporar medições do envelhecimento saudável.

Nesse aspecto, é possível sugerir algumas ações na LC de enfermagem, tais como a implementação de programas de promoção à saúde e prevenção de agravos em nível primário, destacando a importância do envolvimento da família nessas ações. Podem contribuir para um envelhecimento bem-sucedido e sustentável a prestação de cuidados de enfermagem centrados na pessoa em diversas áreas de cuidado e o reforço sobre a importância da avaliação da integralidade do cuidado em todas as áreas - por exemplo, realização de avaliações multidimensionais.

Salienta-se que pesquisas desenvolvidas por enfermeiros relacionadas a temáticas como o autocuidado e a avaliação do cuidado integral podem favorecer o direcionamento de estratégias mais efetivas para o bem-estar da população idosa.

### Promover o acesso aos cuidados de longa duração para as pessoas idosas

A quarta área de ação destaca a importância de promover o acesso aos cuidados de longa duração e apresenta conexão com os seguintes ODS: 3. Enfatiza a importância do envelhecimento saudável para manutenção da saúde e bem-estar; 10. Reforça a necessidade de políticas públicas para superar as iniquidades de acesso aos serviços pela população idosa; e 17. Salienta a importância de parcerias com investimentos favoráveis para grupos etários.

As pessoas idosas com idade avançada podem apresentar declínio da capacidade funcional, geralmente manifestada de forma isolada ou concomitante por alterações cognitivas, físicas e sociais, carecendo de cuidados de longa duração. Nesse contexto, a enfermagem lidera a prestação de um cuidado tipificado, integral e centrado na pessoa idosa em diversos cenários, como Instituições de Longa Permanência, centros de reabilitação e domicílio.

Dessa forma, a implementação de programas de promoção da saúde com foco no autocuidado, voltados para a população idosa e suas famílias, e o alinhamento de um plano longitudinal de cuidados integrais são primordiais como ações de aplicabilidade das LC da enfermagem.

Enfim, os enfermeiros desempenham papel vital na educação de pacientes e de suas famílias, bem como nas medidas de promoção e prevenção, pois desenvolvem o trabalho sob um entendimento mais amplo de saúde^(10)^.

Por sua vez, as pesquisas e consequentes publicações são essenciais para que os resultados cheguem à sociedade de maneira geral, incluindo as pessoas idosas. O enfermeiro, nessa proposição, deve atuar como líder na formulação de políticas públicas, propondo cuidado integral nos diferentes níveis de atenção. Isso inclui desde a APS para a pessoa idosa saudável até os cuidados paliativos. Estudos sobre o cuidado integral e longitudinal nos diferentes níveis de atenção podem nortear a equipe de cuidado, visando favorecer a obtenção de acesso por parte das pessoas idosas aos cuidados de longa duração.

Os profissionais de enfermagem são os que estão mais próximos do público. Por essa razão, eles têm capacidade de gerenciar processos de cuidado eficazes. Cabe destacar que, para atingir o envelhecimento saudável e sustentável, o enfermeiro deve atuar junto com a equipe interdisciplinar, em busca de um olhar multidimensional da pessoa idosa no desenvolvimento de ações integrativas.

Contudo, alguns países carecem de uma gestão capaz de implementar mudanças e valorizar a pessoa idosa considerando a particularidade de cada espaço social. Trata-se de uma proposta arrojada. Nesse sentido, as Nações Unidas, a Organização Mundial de Saúde, a Organização Pan-Americana de Saúde, as universidades e os conselhos de classe devem debater o tema e propor mudanças nos papéis dos enfermeiros.

## CONSIDERAÇÕES FINAIS

A Década de Envelhecimento Saudável e os Objetivos do Desenvolvimento Sustentável, considerados sob a perspectiva das concepções da linha de cuidado de enfermagem, permitem refletir sobre como o enfermeiro pode e deve atuar na implementação de estratégias que propiciem um envelhecimento bem-sucedido e sustentável. Portanto, a efetivação das proposições apresentadas nesta análise reflexiva mostra-se como um dos grandes desafios para o desenvolvimento da enfermagem gerontológica.
